# Formation of oriented fishbone-like pores in biodegradable polymer scaffolds using directional phase-separation processing

**DOI:** 10.1038/s41598-020-71581-y

**Published:** 2020-09-02

**Authors:** Young Gun Ko

**Affiliations:** Department of Chemical Engineering and Materials Science, Sangmyung University, Hongjimun 2-gil 20, Jongno-gu, Seoul, 03016 Republic of Korea

**Keywords:** Biotechnology, Materials science

## Abstract

The scaffold is a dreamed biomaterial of tissue engineers which can culture cells three-dimensionally outgrowing the two-dimensional cell culture in a petri dish to repair or regenerate tissues and organs. To maximize the performance of this dreamed material, complex three-dimensional (3D) structures should be generated with a simple technique and nontoxic ingredients. Many tissues have tubular or fibrous bundle architectures such as nerve, muscle, tendon, ligament, blood vessel, bone and teeth. The concept of mimicking the extracellualr matrix in real tissue has recently been applied to scaffold development. In this study, a novel method for preparing the poly(l-lactic acid) (PLLA) scaffold with a tubular architecture is presented. Solid–liquid phase-separation was applied to form tubular pores in the scaffold using the directional freezing apparatus. Pores formed in this manner exhibited a fishbone like morphology due to the two crystalline phases of 1,4-dioxane. A tubular diameter of ca. 60–250 μm was achieved by regulating the PLLA concentration and the cooling rate. The compressive modulus of the fishbone-like porous scaffold showed higher values than that of non-directional porous scaffold.

## Introduction

Scaffolds are biomaterials designed by tissue engineers to support three-dimensional tissue formation to facilitate repair and regeneration of damaged tissues and organs. In these scaffolds, biodegradable polymer scaffolds are particularly prevalent because over time the artificial scaffold and be completely replaced by the patient’s own cellular material. Currently, the design and fabrication of synthetic biodegradable scaffolds is driven by two materials categories: (1) biodegradable and bioresorbable polymers, which have been effectively used for clinically established products, including polyglic acid (PGA)^1^, poly (l-lactic acid) (PLLA)^2^, poly (d,l-lactic acid) (PDLLA)^3^, and polycaprolactone (PCL)^4^; (2) novel di- and tri-block copolymers which predominantly incorporated PGA, PLA, and PCL in different chain arrangements which confer both degradation and mechanical property customization^5^. Various techniques such as salt leaching^6^, fibrous fabric processing^7^, woven fabric processing^8^, gas forming^9^, emulsion freeze-drying^10^, electro spinning^11^, three dimensional printing^12^, and phase separation^13^ have been developed to fabricate porous biodegradable polymer scaffolds. In addition, various techniques for the fabrication of biodegradable polymeric scaffolds have been tried recently to increase the strength and the antibacterial activity of scaffolds^14–16^.

The concept of mimicking the extracellualr matrix (ECM) in real tissue has been applied to develop scaffolds at macro- and nano-levels. In this study, the microtubular PLLA scaffolds were fabricated to mimic the macro-level morphology of the ECM because many organs and tissues have tubular or fibrous bundle architectures such as nerve, muscle, tendon, ligament, blood vessel, bone and teeth. Solid–liquid phase separation was used to fabricate tubular scaffold. This technique can be achieved by lowering the temperature to induce solvent crystallization from a polymer solution. In this process, pores are created in the scaffold as the solvent crystals are removed through freeze-drying. To create pores with a specific orientation, the solvent in polymer solution was crystallized from bottom to top of the solution-filled mold using the directional freezing apparatus designed in this study. Using this process and 1,4-dioxane as the solvent, scaffolds were formed which exhibited fishbone-like pores. Here, we demonstrate the control of the diameter of microtublular pores and mechanical properties with the cooling rate and compositional values. The fabricated tubular scaffold showed higher compressive modulus values in comparison with the non-directional scaffold when the load was applied parallel to the tubular axis.

## Materials and methods

### Materials

Poly(l-lactic acid) (PLLA) with inherent viscosity of approximately 1.6 dl/g was purchased from Boehringer Ingelheim (Ingelheim, Germany). The PLLA was dissolved in 1,4-dioxane (Aldrich, Milwaukee) to form solutions with desired concentrations (2.5–10 wt/vol%).

### Fabrication of non-uniaxial Porous Scaffolds and Oriented Porous Scaffolds

To fabricate non-directional porous PLLA scaffolds, the polytetrafluoroethylene (PTFE) mold containing PLLA solution was transferred into a freezer at − 20 °C to induce solid–liquid phase separation. The phase-separated PLLA/1,4-dioxane systems were then transferred into a freeze-drying vessel at – 5 to − 10 °C in an ice/salt bath, and freeze-dried under vacuum (pressure lower than 0.5 mmHg) for 2 weeks.

**Figure 1:**
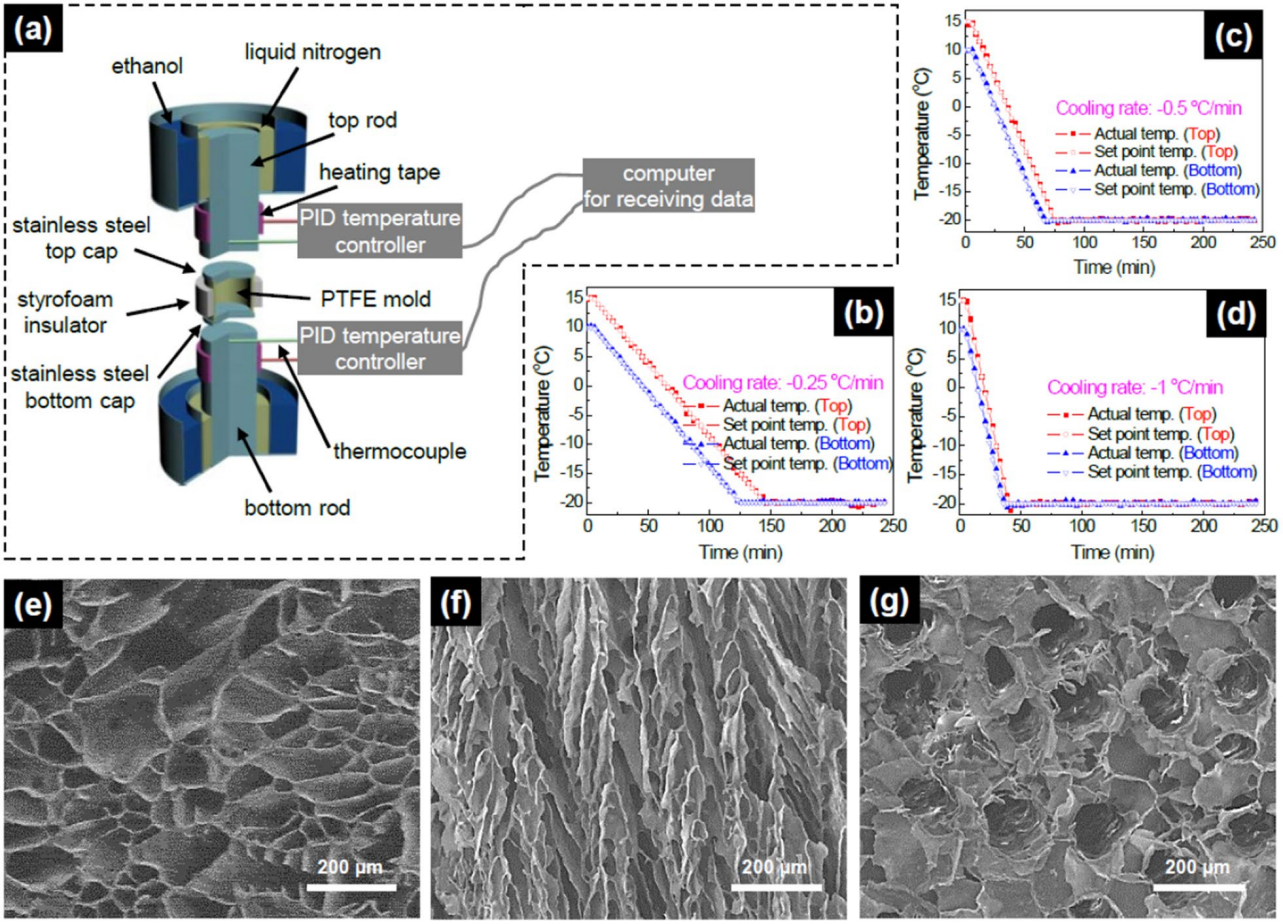
(**a**) Schematic of the apparatus to directionally freeze the PLLA solution. Freezing temperature–time profile (cooling rate) of two rods; (**b**) − 0.25 °C/min, (**c**) − 0.5 °C/min, and (**d**) − 1 °C/min. SEM images of (**e**) non-uniaxial porous PLLA scaffold fabricated in − 20 °C freezer, (**f**) longitudinal section of oriented porous PLLA scaffold, and (**g**) cross section of oriented porous PLLA scaffold.

The oriented tubular scaffold was fabricated by pouring the PLLA solution into the PTFE mold whose top and bottom caps were made of stainless steel (Fig. 1a). That mold was surrounded with the styrofoam insulation and placed between two stainless steel rods. The two rods were immersed in a bath of liquid nitrogen to freeze the mold. The bath of liquid nitrogen was placed in a bath of ethanol to reduce the volatilization of the liquid nitrogen. A computer, PID temperature controllers, and heating tape were used to control the temperature of the stainless steel rods (Fig. 1b–d). The two rods were cooled to − 20 °C with 5 °C temperature gap between top and bottom rods to consider the thermal conduction from bottom to top of the PLLA solution in the mold. After freezing the mold, that was then transferred into a freeze-drying vessel at – 5 to − 10 °C in an ice/salt bath, and freeze-dried under vacuum (pressure lower than 0.5 mmHg) for 2 weeks.

### Characterization

The morphologies of the oriented and non-oriented structures were examined using a field emission gun scanning electron microscopy (FEG SEM) (XL30FEG, Philips) at 15 kV. The specimens were cut by a razor blade, and the exposed surfaces were examined. For microstructural observation, the specimens were coated with gold using a sputter coater (Desk-II, Denton Vacuum Inc.). The gas pressure was less than 50 mtorr; the current was approximately 40 mA; and the coating time was 120 s.

The compressive mechanical properties of the scaffolds were measured with MTS mechanical tester (Synergie 200, MTS Systems Corporation, Cary). Disk specimens with a thickness of 4.15 mm were compressed with a crosshead speed of 0.5 mm/min. The load was applied parallel to the tubular axis. The compressive modulus was determined from the initial linear region of the stress–strain curve.

## Results and discussion

Phase-separation occurs when a homogeneous multi-component system becomes thermodynamically unstable under certain conditions and tends to separate into a multi-phase system in order to lower the system free energy^17^. Especially, solid–liquid phase separation can be achieved by lowering the temperature to induce solvent crystallization from a polymer solution. After the removal of the solvent crystals, the space originally taken by the solvent crystals becomes pores. In this study, 1,4-dioxane was used to form pores in the PLLA scaffold using solid–liquid phase-separation.

Figure 1a is a schematic of the apparatus to directionally freeze the PLLA solution. The oriented pore architectures in the scaffold were achieved by freezing the PTFE mold filled with the PLLA/1,4-dioxane solution between two rods whose temperatures were controlled using liquid nitrogen and heating tape. To control the cooling direction from the bottom to the top of the mold, stainless steel caps were used and the PTFE mold was surrounded with styrofoam insulation. Phase-separation of the PLLA solution was carried out at cooling rate of − 0.25 °C/min, − 0.5 °C/min, and − 1 °C/min (Fig. 1b–d). Square, circle, triangle, and reverse triangle indicate the actual temperature of top rod, the set temperature of the top rod, the actual temperature of the bottom rod, and the set temperature of the bottom rod, respectively. The gap between the actual and the set temperatures did not exceed 0.7 °C.

Figure 1e shows a SEM image of a PLLA scaffold which was fabricated with the non-uniaxial temperature gradient. The pore architecture in the PLLA scaffold was randomly oriented, and pores were not well connected. This non-connected porous architecture can not be used as a scaffold because cells can not penetrate and migrate into the inside of the scaffold to form tissue. When a temperature gradient was maintained uniaxially during the phase-separation process using the directionally freezing apparatus, the fishbone-like pore architecture formed in the PLLA scaffold as shown in Fig. 1f,g. Figure 1f,g show the longitudinal and cross section of the scaffold, respectively. All scaffolds in Fig. 1 were fabricated at the concentration of 5 wt/vol% PLLA and the oriented porous scaffold was fabricated with directionally freezing the PLLA solution filled mold at − 1 °C/min of cooling rate.

**Figure 2:**
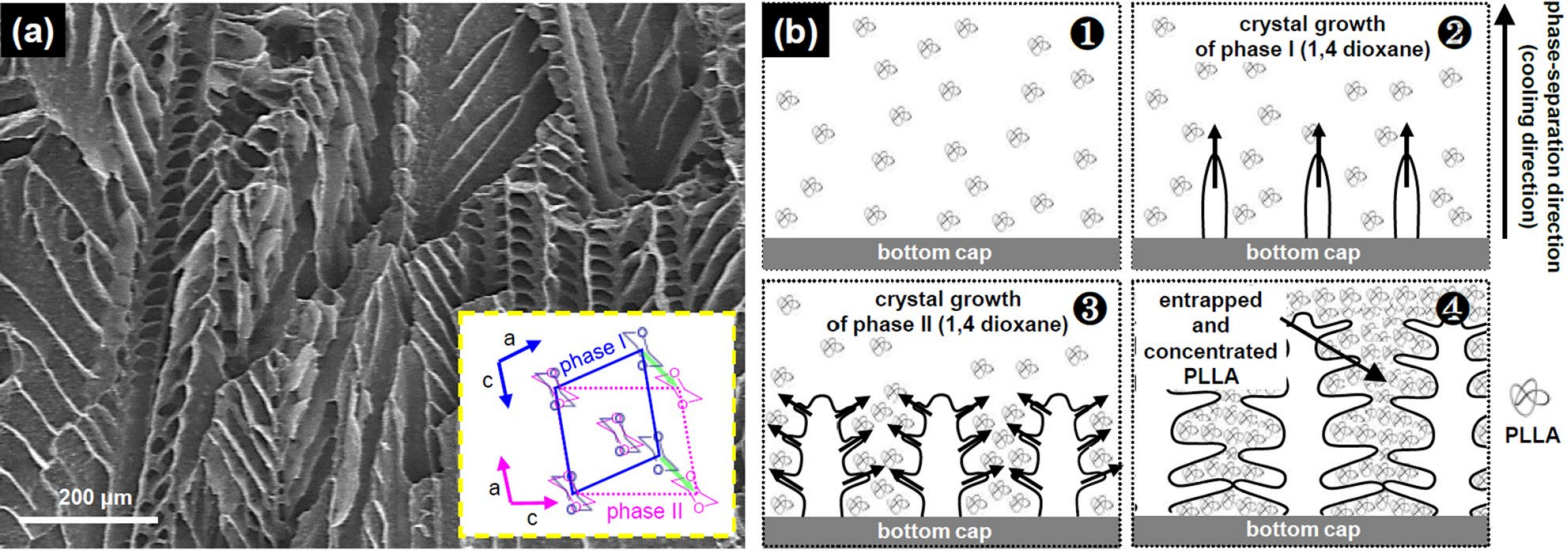
(**a**) A SEM image of longitudinal section of oriented porous PLLA scaffold (inset: two crystal phases of 1,4-dioxane). (**b**) Processing principle on formation of fishbone-mimic pores in PLLA scaffold. The time sequence of the processing principle follows Arabic numeral numbers.

A SEM image of a PLLA scaffold fabricated at 2.5 wt/vol% of PLLA concentration and − 1 °C/min of cooling rate using the directional-freezing apparatus is shown in Fig. 2a. Interestingly, oriented fishbone-like pores formed in the PLLA scaffold. In the technique of solid–liquid phase-separation, the pore shape in the scaffold follows the shape of the solvent crystal. 1,4-dioxane, which is used as a solvent to form pores in the PLLA scaffold, has two crystalline phases^18^. Phase I exists in the temperature region from 5 to 12 °C while phase II exists in the temperature region from – 140 to 5 °C. Although both phases are monoclinic, they have different values of crystal lattice and show the different direction of crystal growth. When temperature range of phase I changes to that of phase II, it appears that both crystal cells are projected down the monoclinic *b* axis as shown in Fig. 2a (inset). The freezing process of PLLA/1,4-dioxane solution using the directional freezing apparatus was conducted within the temperature range of 15 to − 20 °C. In this temperature range, both crystal phases of 1,4-dioxane formed together. Above the temperature of 5 °C, only crystalline phase I of 1,4-dioxane forms. However, below 5 °C, crystalline phase II of 1,4-dioxane also forms at the low region of crystallized phase I because the temperature of the high region of crystallized phase I is higher than the temperature of the formation of crystalline phase II of 1,4-dioxane. Figure 2b shows the processing principle on the formation of fishbone-like pores in PLLA scaffold. As the temperature decreases at the bottom cap, the crystalline phase I of 1,4-dioxane grows at the surface of the bottom cap. And as the temperature decreases further, the crystalline phase II of 1,4-dioxane grows on the already formed crystalline phase II of 1,4-dioxane like branches. The entrapped and concentrated PLLA between 1,4-dioxane crystals solidifies after the removal of 1,4-dioxane using the freezing-dryer. The spaces created by the two phases of 1,4-dioxane crystals become pores that exhibit a fishbone-like morphology.

**Figure 3:**
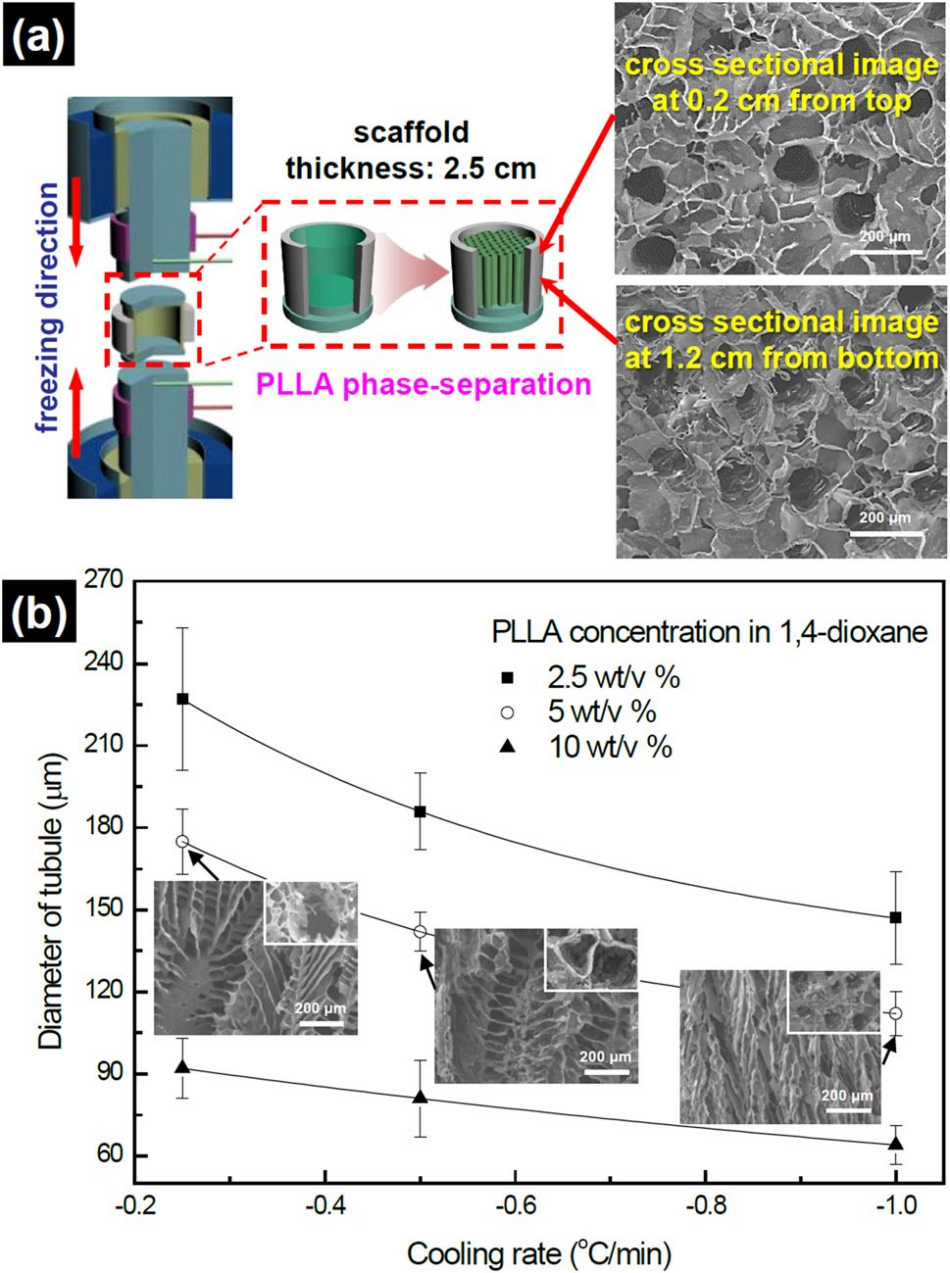
(**a**) Cross sectional SEM images from different locations of the scaffold fabricated with 5 wt/vol% PLLA at the cooling rate of − 1 °C/min. (**b**) Diameter of fishbone-mimic pore in PLLA scaffold. Each SEM images in the graph shows the longitudinal and cross (insets) sections of scaffold fabricated at the indicated condition.

In order to adapt these novel scaffolds to various tissue types, it was necessary to develop methods whereby the pore architecture could be controlled. Various diameters of tubular pores are required because tubular or fibrous bundle architectures in organs and tissues have different diameters of tubules to perform their characteristic functions; for example, ca. 90–260 μm tubular diameter of osteon in the compact bone^19^ as compared to ca. 5–100 μm diameter of skeletal muscle fiber^20^. In the previous study, oriented-tubular scaffolds were manufactured successfully by simply placing the polymer solution filled mold on the top of a metal block in a freezer^21^. However, the diameter of microtubules was smaller than 120 μm. The cooling rate for the phase-separation was not controlled. Therefore, the diameter of microtubules was controlled only by adjusting the polymer concentration. In this study, the diameter of tubular pores was regulated with the control of the PLLA concentration and the cooling rate together. The temperature gradient in the fabrication mold makes the pore size gradient in the fabricated scaffold. Therefore, temperatures of top and bottom cooling rods decreased with a temperature gap to remove or reduce the sudden temperature gradient in the mold (Fig. 1b–d). The pore size at various locations of the fabricated scaffold were investigated using SEM, and the pore size gradient for the scaffold was not observed. All pore sizes were same regardless of location in the scaffold. Cross sectional SEM images at top and middle of the fabricated scaffold were shown in Fig. 3a. Increasing the PLLA concentration reduced the diameter of the tubules. This point is made clear by the comparison between two scaffolds which were fabricated with 2.5 wt/vol% and 10 wt/vol% at the cooling rate of − 0.25 °C/min. The scaffold fabricated with 2.5 wt/vol%, showed larger diameter than the scaffold fabricated with 10 wt/vol%, by ca. 2.5 times as shown in Fig. 3b. In all cases, increasing the concentration of the PLLA solution reduced the diameter of microtubules. The relationship between the tubular diameter and the concentration of PLLA solution can be considered as follows; high PLLA concentration can produce more PLLA tubules in comparison with low PLLA concentration. Therefore, the tubular diameter of the scaffold fabricated at high PLLA concentration became smaller than that of the scaffold fabricated at low PLLA concentration as shown in Fig. 3b. Figure 3b also shows that reducing the cooling rate increased the diameter of the tubular pores. In the case of the concentration of 2.5 wt/vol% PLLA, the scaffold, which was fabricated at the cooling rate of − 0.25 °C/min, exhibited pores with a larger diameter than the scaffold, which was fabricated at − 1 °C/min, by ca. 1.5 times. As previously stated, the space originally taken by the solvent crystals becomes pores. A low cooling rate along *c* axis allows for increased crystallization along the *a* and *b* axes. Therefore, the expansion along *a* and *b* axes of the 1,4-dioxane crystal creates a larger pore in the scaffold. By controlling the PLLA concentration and the cooling rate together, the diameter range of ca. 60–250 μm were fabricated as tubular pores in the scaffold.

**Figure 4:**
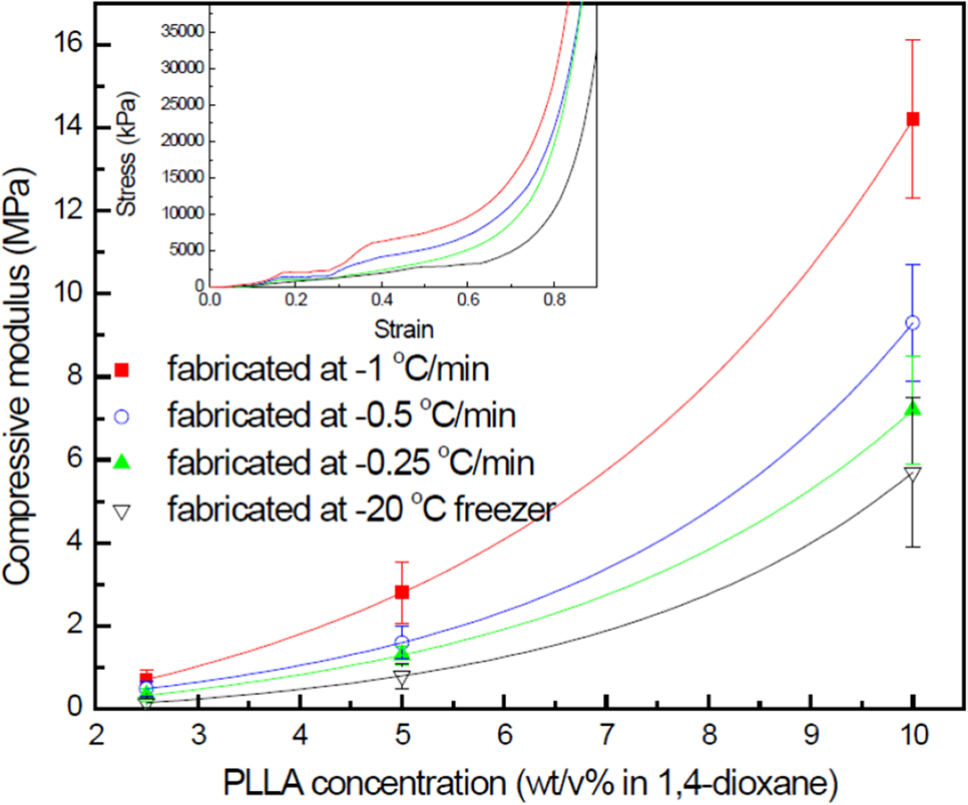
Compressive modulus of PLLA scaffolds against vertical loading (inset: strain–stress curves for 10 wt/vol% PLLA scaffolds).

Ideally, scaffolds used for the regeneration of tissues and organs should have a constant morphology and the optimum strength matching the performance of the native tissues and the organs. Unfortunately, PLLA has limited strength although it is highly biodegradable. For this reason, the use of architectures which improve the mechanical properties of PLLA scaffolds is of interest. In nature, oriented porous structures such as a compact bone^22^, a cedar tree^23^, a grassy stem^23^ and a blue jay feather rachis^23^ are observed easily and show high modulus in spite of their low density. The compressive modulus of the fishbone-like porous scaffold showed higher values than that of non-directional porous scaffold as shown in Fig. 4. In the case of the concentration of 10 wt/vol% PLLA, the microtubular scaffold, which was fabricated at the cooling rate of − 1 °C/min, showed higher compressive modulus value than the non-directional scaffold, which was fabricated at − 20 °C freezer, by ca. 5 times. Notably, the fabricating condition of fishbone-like porous scaffolds at the high PLLA concentration and the low cooling rate produced the scaffold with the highest modulus. These conditions created scaffolds with smaller diameter and more numerous tubules. This phenomenon can be explained by the concept of energy dissipation during compression. Scaffolds with smaller but more numerous tubules are able to withstand greater compressive forces due to the improved distribution of the load between tubules. The relationship and the related theory between osteons’ diameter and fracture energy was reported by other research groups and indicated that smaller and more numerous osteons shows increased strength^24–26^.

## Conclusions

Oriented fishbone-like pores in the poly (l-lactic acid) (PLLA) scaffold were successfully fabricated with 1,4-dioxane using the directional freezing apparatus. Crystalline two phase of 1,4-dioxane formed at different temperature range during the freezing process, and then frozen crystals of 1,4-dioxane were removed from PLLA solution using the freeze-dryer. By this process, PLLA scaffold exhibiting fishbone-like pores were fabricated. The diameter of tubular pores was regulated (within the range of ca. 60–250 μm) through controlling the PLLA concentration and the cooling rate. High PLLA concentration and a slow cooling rate produced scaffolds with the largest tubular diameter. The compressive modulus of the fishbone-like porous scaffold showed higher values than that of non-directional porous scaffold. The fabricating condition of fishbone-like porous scaffolds at the high PLLA concentration and the low cooling rate greatly improved the mechanical properties of the scaffold. We expect that the developments presented in this paper will gain wide ranging applications in many areas; such as scaffolds for tissue engineering, porous membranes, multi-channel processes for chemical reaction, and packaging materials.
